# *CREBBP* and *EP300* mutational spectrum and clinical presentations in a cohort of Swedish patients with Rubinstein–Taybi syndrome

**DOI:** 10.1002/mgg3.177

**Published:** 2015-09-22

**Authors:** Josephine Wincent, Aron Luthman, Martine van Belzen, Christian van der Lans, Johanna Albert, Ann Nordgren, Britt‐Marie Anderlid

**Affiliations:** ^1^ Department of Molecular Medicine and SurgeryCenter for Molecular MedicineCMM L8:02Karolinska InstitutetKarolinska University HospitalStockholmSweden; ^2^ Department of Clinical GeneticsLeiden University Medical CenterLeidenThe Netherlands; ^3^ Division of SurgeryDepartment of Clinical ScienceKarolinska InstitutetDanderyd HospitalStockholmSweden; ^4^ Department of Clinical GeneticsKarolinska University HospitalStockholmSweden

**Keywords:** *CREBBP*, delayed emergence after anesthesia, *EP300*, preeclampsia, Rubinstein‐Taybi syndrome

## Abstract

Rubinstein–Taybi syndrome (RTS) is a rare autosomal dominant congenital disorder characterized by distinctive facial features, broad thumbs and halluces, growth retardation, and a variable degree of cognitive impairment. *CREBBP* is the major causative gene and mutations in *EP300* are the cause of RTS in a minority of patients. In this study, 17 patients with a clinical diagnosis of RTS were investigated with direct sequencing, MLPA, and array‐CGH in search for mutations in these two genes. Eleven patients (64.7%) had disease‐causing point mutations or a deletion in *CREBBP* and in one patient (5.9%) a causal *de novo* frameshift mutation in *EP300* was identified. This patient had broad thumbs, mild intellectual disability, and autism. In addition, an inherited missense mutation of uncertain clinical significance was identified in *EP300* in one patient and his healthy father, and three patients had intronic nucleotide changes of uncertain clinical significance in *CREBBP*. Snoring and sleep apnea were common in both groups and four of the patients' mothers had preeclampsia during pregnancy. Importantly, difficulties associated with anesthesia were frequently reported and included delayed or complicated emergency in 53.3% of patients.

## Introduction

Rubinstein–Taybi syndrome (RTS [OMIM 180849]) is an autosomal dominant congenital disorder with an estimated incidence of 1:100,000–125,000. RTS is characterized by distinctive facial features (downward slanting palpebral fissures, arched eyebrows, beaked nose, columella below alae nasi and micrognathia), skeletal abnormalities (typically broad thumbs and halluces), growth retardation, microcephaly, and a variable degree of cognitive impairment and behavioral problems (Roelfsema and Peters 2007; Schorry et al. 2008). A few familial cases demonstrating germline and somatic mosaicism have been reported (Chiang et al. 2009; Bartsch et al. 2010a).

Around 10% of the patients with RTS have microdeletions of chromosome 16p13.3 involving *CREBBP*, and in approximately 50% of the patients point mutations in *CREBBP* are found (Schorry et al. 2008). There is no clear genotype–phenotype correlation (Schorry et al. 2008). Mutations in *EP300* are the cause of RTS in a minority of patients, and to the best of our knowledge only 16 patients have been described to date (Roelfsema et al. 2005; Bartholdi et al. 2007; Zimmermann et al. 2007; Foley et al. 2009; Bartsch et al. 2010b; Tsai et al. 2011; Woods et al. 2014; Negri et al. 2015; Solomon et al. 2015). Eleven additional mutations are listed in the database LOVD (http://chromium.liacs.nl/LOVD2/variants.php?action=search_all). *CREBBP* and *EP300* are ubiquitously expressed and are highly homologous genes. *CREBBP*, a 150 kb gene with 31 exons, encodes the 2442 amino acid CREB‐binding protein. *EP300* is located on chromosome 22q13.2 and its 31 exons encode the E1A‐1‐binding protein p30, consisting of 2415 amino acids. The two proteins act as transcriptional coactivators by forming scaffolds between the RNA polymerase II complex and DNA‐binding transcription factors. They also affect gene expression by serving as histone acetyltransferases (HATs). There are, however, subtle differences between these two proteins and their expression pattern during embryogenesis is not completely overlapping (Roelfsema and Peters 2007).

In this study, we report the mutation spectrum in *CREBBP* and *EP300* among 17 patients with a clinical diagnosis of RTS, including a novel pathogenic *EP300* mutation and intronic *CREBBP* alterations of uncertain clinical significance, together with detailed clinical phenotypes.

## Materials and Methods

### Patients

Seventeen patients with a clinical diagnosis of RTS were included after obtaining informed consent and the study was performed with approval of the regional ethics committee at the Karolinska Institutet, Stockholm. All the patients were examined by clinical geneticists at the clinical genetics department at the Karolinska University Hospital. The Genomic DNA from the patients and their parents, when available, was isolated from peripheral blood according to the standard procedures. Clinical data is summarized in Table 1.

**Table 1:** Clinical data

Patient	1	2	3	4	5	6	7	8	9	10	11	12	13	14	15	16	17
Mutation	Del^a^	Ns^a^	Ns^a^	Ss^a^	Fs^a^	Ns^a^	Ns^a^	Ns^a^	Fs^a^	Ms^a^	Ns^a^	Fs^b^	Ms^b^				
Sex	F	M	F	M	M	M	F	M	F	F	F	M	M	M	F	M	F
Age (diagnosis/examined)	1 m/32 y	17 y/21 y	13 y/13 y	n.i./16 y	2 y/2 y	1 y/17 y	n.i./4 y	2 m/12 y	1 y/18 y	2 y/10 y	4 y/17 y	2 y/5 y	1.5 y/5 y	11 m/13 y	12 y/18 y	1 y/1 y	9 y/14 y
Mat. preeclampsia	−	+	−	−	n.i.	n.i.	n.i.	+	−	+	−	+	−	−	−	n.i.	−
Gestation week	40	35	41	40	41	n.i.	n.i.	42	38	40	n.i.	38	39	42	37	37	41
Birth weight (g)	3200	2100	3175	2760	3570	n.i.	n.i.	4150	2635	2340	n.i.	2780	3625	3145	2800	n.i.	2980
Birth length (cm)	48	45	47	46	n.i.	n.i.	n.i.	52	47	47	n.i.	47	51	49	48	n.i.	50
Postnatal length	−4 SD	−7 SD	−2 SD	n.i.	−1.5 SD	−5 SD	−1 SD	−2 SD	−3.5 SD	−2 SD	−2 SD	−3 SD	−1.5 SD	−3.5 SD	−6 SD	−3.5 SD	−1 SD
Microcephaly	+ (−3.5 SD)	+	+	+	+ (−4 SD)	− (−2 SD)	−	−	− (−2 SD)	+ (−3.5 SD)	− (−1.5 SD)	+ (−4 SD)	− (−1 SD)	− 2.5 SD	+ (−4 SD)	−	− (−1 SD)
Feeding difficulties	+	++	++	+	n.i.	+	n.i.	+	++	+	+	−	+	+	++	+	+
Hypotonia	+	+	−	+	−	+	n.i.	+	−	−	−	−	+	+	+	+	+
Hirsutism	+	−	+	n.i.	n.i.	+	+	n.i.	+	+	+	−	n.i.	+	+	+	+
Beaked nose/columella below alae nasi	+/+	+/+	+/+	+/+	n.i./n.i.	+/+	+/+	+/+	+/+	+/+	+/+	+/+	+/+	n.i.	+/+	n.i.	n.i.
Down‐slanting palpebral fissures	+	+	+	+	+	+	+	+	+	+	+	+	+	+	+	n.i.	+
Long eye lashes/Arched eyebrows	+/+	+/+	+/+	+/+	n.i./n.i.	+/+	+/+	+/+	+/+	+/+	+/+	+/−	+/+	+/+	+/+	+/n.i.	+/n.i.
Micrognathia	−	+	+	+	n.i.	+	+	−	−	+	+	+	+	+	+	+	n.i.
Highly arched palate	+	+	+	+	+	+	+	n.i.	+	+	+	+	+	+	+		+
Broad thumbs/halluces	+/+	+/+	+/+	+/+	+/+	+/+	+/+	+/+	+/+	+/+	+/+	+/+	+/+	+/+	+/+	+/+	+/+
Radially deviated thumbs	+	−	−	−	n.i.	+	+	−	−	−	−	−	+	−	−	n.i.	n.i.
Heart/Urogenital malformation	n.i./−	+/+	−/−	−/+	−/+	+/+	+/−	+/+	−/+	−/−	−/−	+/+	−/+	−/+	−/+	+/+	−/−
Keloids/Tumors	−/−	−/−	+/−	+/−	+/n.i.	+/−	−/n.i.	+/n.i.	+/−	−/−	+/−	−/−	−/−	+/−	+/n.i	+/n.i.	−/−
Impaired hearing/vision	−/+	−/−	+/+	−/+	−/+	−/+	n.i./−	−/+	+/+	−/+	−/+	−/−	−/−	−/+	−/+	−/+	+/−
Kyphosis/Scholiosis	+/−	+/+	+/+	+/−	−/−	+/−	−/−	+/+	+/−	−/−	−/−	−	−	−/−	−/+	n.i./n.i.	−/−
Epilepsia	−	+	−	−	−	−	n.i.	+	−	−	+	−	−	−	+	−	−
Speech delay	+	+	+	+	+	+	+	+	+	+	+	+	+	+	+	+	+
Intellectual disability	Mild	Severe	Severe	Moderate	Mild	Moderate	+	+	Mild	+	Severe	Mild	Mild	Moderate	+	Moderate	Moderate
Behavior																	
Sociable/Autism	−/−	+/+	n.i./n.i.	+/−	n.i./n.i.	+/n.i.	n.i./n.i.	+/n.i.	+/−	−/n.i.	−/+	−/+	+/−	−/+	+/+	n.i./n.i.	−/n.i.
Anxiety/Aggression	−/−	−/−	+/+	+/−	n.i./n.i.	−/−	n.i./n.i.	n.i./n.i.	+/−	n.i./+	−/−	+/+	−/−	−/−	+/−	n.i./+	+/+
Short attention span	−	+	n.i.	−	n.i.	−	n.i.	+	−	+	−	−	−	+	−	n.i.	−
Recurrent infections	+	−	+	−	n.i.	+	+	+	+	+	+	+	+	−	+	n.i.	−
Constipation	+	+	−	−	n.i.	−	n.i.	+	+	+	+	−	−	+	+	−	+
Sleep apnea/snoring	−/+	−/−	+/+	−/+	+/+	+/+	n.i./n.i.	+/n.i.	+/+	−/−	−/−	−/−	+/+	+/+	+/+	n.i./n.i.	n.i./n.i.
Complicated emergence after anesthesia	+	+	n.i.	−	+	−	−	+	+	−	+	−	−	−	+	+	−

Del, Deletion; Ns, Nonsense; Ss, Splice site; Ms, Missense; n.i., no information available; m, month; y, year.

a *CREBBP*.

b *EP300*.

### Clinical information of patients 2 (foot malformation), 12 and 13 (*EP300* mutations)

**Figure 1:**
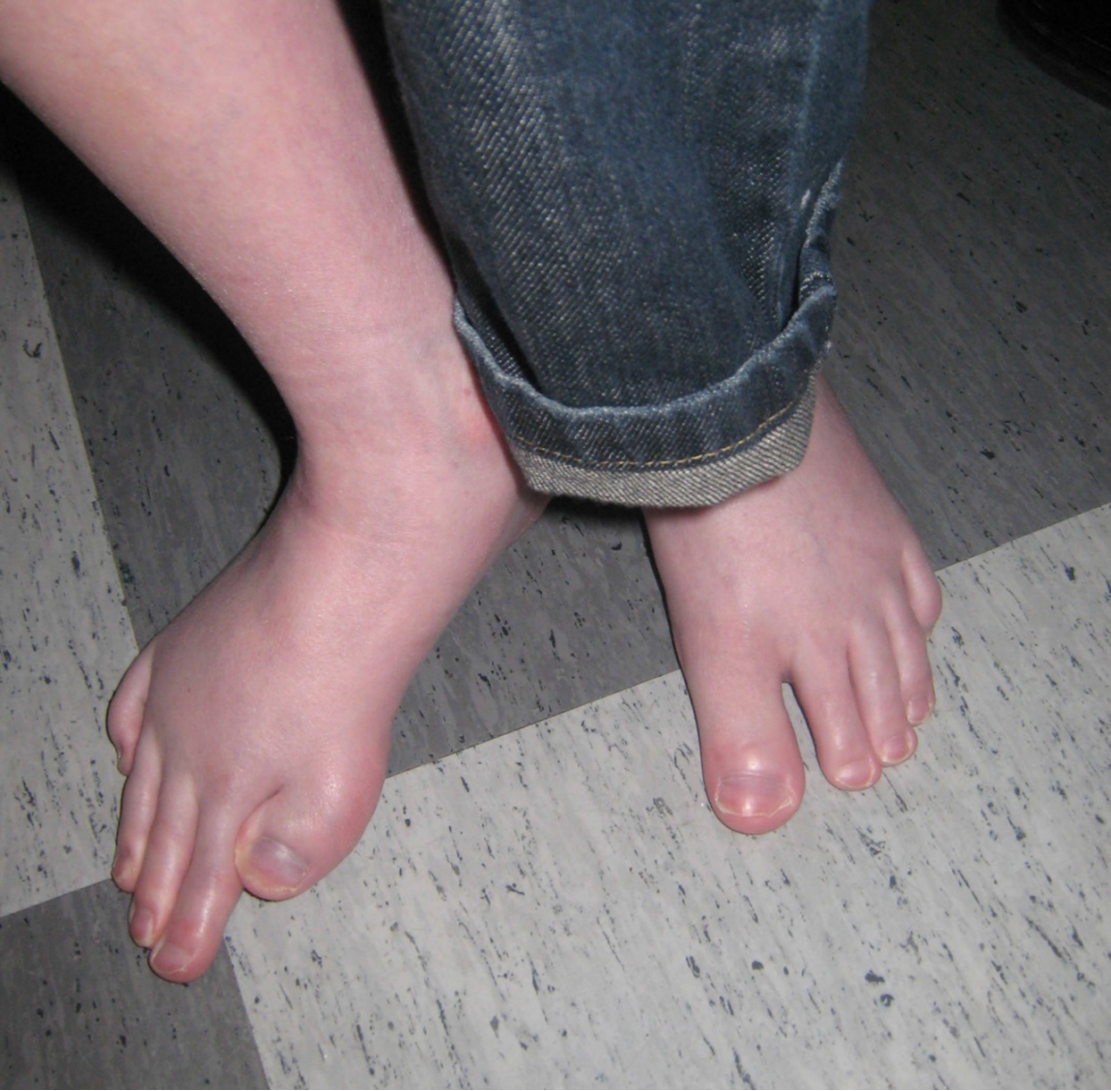
Picture of patient 2's feet. Note broad hallux and long toes on the left foot, broad laterally deviating hallux and extremely long toes on the right foot.

Patient 2 was the third child to healthy nonconsanguineous parents. There was no family history of malformations or intellectual disability. The boy was born at gestation week 35 due to premature rupture of membranes. The birth weight was 2100 g (−2 SD) and birth length 45 cm (−1 SD). In the neonatal period, muscular hypotonia, bilateral cryptorchidism, breathing difficulties, broad thumbs and halluces, syndactyly IV and V, and a hemangioma in the forehead was noted. He started walking at an age of 3.5 years but even at an older age he needed a wheelchair for longer transportations. The boy had no spoken language but communicated with approximately 25 signs. He had severe ID, short stature, microcephaly, and facial appearance characteristic for RTS. In addition he had a delayed maturation of the skeleton, severe scoliosis, and an unusual foot malformation (Fig. 1).

Patient 12 was the first child born to healthy nonconsanguineous parents. The mother, however, had broad thumbs. The parents have had five miscarriages. The mother had high blood pressure during the pregnancy and labor was induced due to preeclampsia. The boy was born at gestation week 38 + 3. The birth weight was 2780 g (−1.5 SD), birth length 47 cm (−1.6 SD), and head circumference (HC) 33 cm (−1.5 SD). At an age of 2 years the HC had decreased to −5 SD and at age 5 years the height and weight had decreased to −3 SD and −2.5 SD, respectively. In addition to microcephaly, he had arched eyebrows, long eyelashes, columella below alae nasi, downward slanting palpebral fissures, epicanthal folds and a highly arched palate. He had broad thumbs, clinodactyly of digit V, and broad halluces. He had a ventricular septal defect and cryptorchidism. He walked independently at an age of 20 months and had a mild developmental delay. There was a speech delay with the first spoken words at an age of 24 months, with a better understanding then expressive language. Furthermore he developed behavioral problems including self‐mutilation and daily temper tantrums. Other observed anomalies included a short plica aryepiglottica and frequent pneumonias.

Patient 13 was the second child to healthy consanguineous parents. A paternal uncle had intellectual disability. The boy was born at gestation week 39 + 3 after a normal pregnancy. The birth weight was 3625 g (M), birth height 51 cm (M) and HC 35 cm (M). At an age of 6 years there was no growth retardation or microcephaly (although HC had decreased to −1.5 SD). He had muscular hypotonia, walked independently at an age of 2 years, spoke his first words at an age of 2 years and had a cognitive developmental delay. He had dysmorphic facial features including arched eyebrows, long eyelashes, low hairline, columella below alae nasi, downward slanting palpebral fissures, and low set ears. He had broad thumbs with radial angulation, broad halluces and pectus excavatum. He had frequent otitis and underwent tonsillectomy.

### Array‐CGH

Array comparative genomic hybridization was performed in all the patients except patient 3, 4, 6, and 10 (lack of DNA) to search for microdeletions and microduplications. 244K/180K oligonucleotide arrays with complete genome coverage produced by Agilent Technologies (Palo Alto, CA) or Oxford Gene Technology (Oxford, UK) were used. Experiments were performed according to the manufacturers' protocol. After hybridization and washing, the slides were scanned on an Agilent Microarray Scanner and captured images were analyzed with Feature Extraction Software v.9.113 (Agilent Technologies) and DNA analytics v. 4.0 or Cytosure Interpret Software v.3.3.2 (Oxford Gene Technologies). A threshold of at least three consecutive aberrant probes was applied, resulting in an average resolution of approximately 30 kb. Genomic positions are according to the NCBI36/hg18 build in UCSC.

### Sequencing and MLPA

Polymerase chain reaction (PCR) amplification, followed by direct sequencing of the coding sequence and the corresponding exon–intron boundaries of *CREBBP* (GRCh38:CM000678.2) and *EP300* (GRCh38:CM000684.2) was performed according to the standard procedures. Primers used for PCR amplification of CREBBP were previously reported by Coupry et al. (2002), except primers for amplification of exon 1, 2, 18, 27, 30, and 31 for which additional primers were designed in order to improve the readability of the sequences (primer sequences available upon request). Sequencing of the coding regions was performed using Big Dye Terminator cycle sequencing kit 3.1 (Applied Biosystems, Foster City, CA) according to the manufacturer's standard protocol and sequenced on an ABI genetic analyzer. ABI SEQSCAPE software version 2.5 (Applied Biosystems) was used to perform sequence analysis. A search for intragenic deletions and duplications was performed by MLPA. The commercial kits P313 and P333 were used (MRC Holland, Amsterdam, the Netherlands). The MLPA reaction was performed according to the manufacturer's standard protocol and reagents. Data analysis was performed using Microsoft Excel. Analysis of *EP300* was performed in the patients without pathogenic *CREBBP* mutations, but was unfortunately not possible in patients 16 and 17 due to lack of DNA. Parental samples were analyzed for the variants identified in the child (not done for patients 3 and 7).

## Results

The clinical phenotypes of the patients are summarized in Table 1. The patients displayed a classic RTS‐phenotype with characteristic facial features, broad thumbs, and halluces and a variable degree of intellectual disability. Five patients had autism and eight patients had behavioral problems comprising anxiety and/or aggression. Fifty percent had a pronounced short stature (less than −2.5 SD). Patient 2 had a very rare morphology of his right foot (Fig. 1). Ten patients had trouble with snoring and/or sleep apnea (often improved after surgery). Difficulties associated with anesthesia comprising delayed or complicated (apnea, agitation, postoperative breathing difficulties) emergence was reported in 8/15 patients (53.3%) for which information were available. Maternal preeclampsia was reported for three patients with *CREBBP* mutations and one patient with *EP300* mutation.

The *CREBBP* and *EP300* mutations identified in our cohort of 17 Swedish patients with a clinical diagnosis of RTS are summarized in Table 2. Ten patients (58.8%) had causal point mutations in *CREBBP*, comprising six nonsense mutations, two frameshift mutations, one missense mutation and one splice‐site mutation. Eight of the mutations were found to be *de novo* after testing of both parents, but for two of the patients (3 and 7) DNA samples for both parents were not available and the inheritance pattern was thus unknown (although the mother was excluded as a carrier in patient 3). One patient (5.9%) had a 240 kb deletion of chromosome 16p13.3 including the first two exons of *CREBBP*. In *EP300,* one (5.9%) likely causal *de novo* frameshift mutation was identified in patient 12 and an inherited missense mutation of uncertain clinical significance was identified in patient 13.

**Table 2:** *CREBBP* and *EP300* mutation spectrum

Patient	Exon	Nucleotide change	Amino acid change	Inheritance	Controls
*CREBBP* pathogenic mutations					
1	1–2	Del (chr16:3818420‐ 4057031), 240 kb		n.i.	
2	2	c.778C>T	p.Gln260X	*de novo*	
3	4	c.1069C>T	p.Gln357X	Mother excluded	
4	9	c.1941+1G>A	p.?	*de novo*	
5	14	c.3014_3015insC	p.Ser938fs	*de novo*	
6	18	c.3452G>A	p.Trp1151X	*de novo*	
7	18	c.3517C>T	p. Arg1173X	n.i.	
8	24	c.4078C>T	p.Arg1360X	*de novo*	
9	27	c.4400_4401insATGT	p.Thr1468fs	*de novo*	
10	28	c.4613C>G	p.Pro1538Arg	*de novo*	190 negative
11	31	c.5635C>T	p.Gln1879X	*de novo*	
*EP300* mutations					
12	30	c.4783_4784delTT	p.Phe1595fs	*de novo*	
13	31	c.5824A>T	p.Met1942Leu	Inherited, healthy father	189 negative
*CREBBP* intronic variants of uncertain clinical significance					
13	25	c.4134‐6T>C	p.?	*de novo*	175 negative
14	15	c.2881‐13G>A	p.?	*de novo*	190 negative
15	21	c.3836+5G>C	p.?	n.i.	175 negative

Three patients had intronic nucleotide changes in *CREBBP* that are not reported as normal variants previously (to the best of our knowledge). Two of the variants were *de novo* and for one patient parental DNA was not available. None of them were found in 175 healthy controls. No intragenic deletions or duplications of *CREBBP* or *EP300* were detected by MLPA (not performed in patient 1).

## Discussion

Patients 1–12 were found to have pathogenic mutations (*de novo*, truncating, and/or previously described as pathogenic). Due to lack of parental DNA, it was not possible to determine if the mutations were *de novo* or not in patients 1, 3, and 7 . However, these mutations are likely to be pathogenic because they either remove the 5′ end of the gene or lead to premature stop codons, and in addition, the mutation found in case 7 had been reported previously (Bentivegna et al. 2006; Roelfsema and Peters 2007). The previously unreported missense mutation (p.P1538R) found in patient 10 was considered pathogenic as it was *de novo*, located in the HAT domain, was not detected in 190 control subjects and was predicted to be probably damaging using the bioinformatics tool PolyPhen (http://genetics.bwh.harvard.edu/pph2/).

Three patients had intronic mutations in *CREBBP* of uncertain clinical significance. The T>C transition (six nucleotides before exon 25) identified in patient 14 was not predicted to introduce a new splice site using the in silico tool Alamut (Alamut v 2.0; Interactive Biosoftware). However, in patient 15, the G>A transition (13 nucleotides before exon 15) was predicted to disrupt the splice site and introduce a new splice site. This change was not detected in parental DNA or in healthy controls making it a good candidate for being the cause of the syndrome in this patient. Also, the G>C transition (5 nucleotides after exon 21) was predicted to disrupt the splice site by Alamut, but in this patient the inheritance is not known. Unfortunately, RNA from these three individuals was not available and further experiments are needed to evaluate the pathogenicity. The relatively low diagnostic yield in RTS might, to a certain degree, be due to the intronic mutations not immediately adjacent to the intron–exon boundaries.

Of the detected *EP300* mutations, the *de novo* frameshift mutation in patient 12 is considered pathogenic while the inherited missense mutation (p.M1942L) found in patient 13 is of uncertain clinical significance. Although the missense mutation was not identified in the healthy control subjects, it was predicted to be benign by polyphen and the patient's father was healthy in contradiction for it being pathogenic. However, a paternal uncle had an unexplained ID (DNA unfortunately not available) and reduced penetrance is another possibility. In addition, one missense mutation (p.N1511T), inherited from a healthy mother and considered not causative, has been previously reported (Negri et al. 2015). Clinical manifestations in 16 previously described patients with *EP300* mutations have frequently comprised a characteristic facial appearance, microcephaly, short stature, broad thumbs, varying degrees of developmental delay, neurocognitive dysfunction, and only occasionally multiple congenital anomalies (Negri et al. 2015; Solomon et al. 2015). While comparing patient 12 with these patients, we can add to the evolving picture of a phenotype comprising broad thumbs but absence of radial deviation of thumbs and halluces, mild DD, and presence of neuropsychiatric traits.

There may be an association with preeclampsia in mothers of patients with RTS. Including our patient, nine (52.9%) of the 17 hitherto reported *EP300* mutation‐positive patients included a maternal history of preeclampsia (Negri et al. 2015; Solomon et al. 2015). However, in our cohort of *CREBBP* mutation‐positive patients, three out of eight mothers had preeclampsia. Our cohort is relatively small and the information was only available for eight of the mothers, but it gives rise to the question if it is rather the general RTS characteristics of the fetus that may be associated with the preeclampsia than specifically the *EP300* mutations.

Rubinstein–Taybi syndrome is a rare syndrome but many children with the diagnosis undergo several operations. Delayed recovery from anesthesia in RTS have been reported previously (Dunkley and Dearlove 1996), although to a lesser extent than in our patient cohort. Therefore, we urge anesthesiologists to be aware of the possibility of delayed recovery and to use sedative agents with caution.

## Conclusions

We here report the *CREBBP* and *EP300* mutation spectrum in a cohort of 17 Swedish patients with a clinical diagnosis of RTS. Our results confirm that mutations in *CREBBP* are the major cause of RTS and we report a novel causative *EP300* mutation. Three patients had intronic findings of uncertain clinical significance in *CREBBP*, of which at least one is a good candidate for being the causative mutation. Preeclampsia was reported in mothers of RTS patients with both *CREBBP* and *EP300* mutations. In addition, anesthesiologists should be aware that there may be a delayed emergence after anesthesia.

## Conflict of Interest

The authors declare no conflict of interest.
