# Erratum

**DOI:** 10.1002/mgg3.218

**Published:** 2016-05-12

**Authors:** Josephine Wincent, Aron Luthman, Martine van Belzen, Christian van der Lans, Johanna Albert, Ann Nordgren, Britt‐Marie Anderlid

The original article to which this Erratum refers was published in *Molecular Genetics and Genomic Medicine* 4(1):39–45 (DOI 10.1002/mgg3.177). In table 2, patient 5, the mutation c.3014_3015insC was reported incorrectly and should instead be c.2810dupC. The data listed under Amino acid change is correct.

We apologize for this error.

